# Cystic pulmonary tuberculosis: A rare form of an ancient disease

**DOI:** 10.1002/rcr2.1020

**Published:** 2022-08-24

**Authors:** Luong Dinh Van, Huy Ngoc Le, Michel Pletschette, Anh Tuan Nguyen, Thinh Hoang Nguyen, Ngoc Bich Thi Nguyen

**Affiliations:** ^1^ Vietnam National Lung Hospital Hanoi; ^2^ University of Munich LMU Medical Center, Dept. of Infectious and Tropical Diseases Munich Germany; ^3^ Radiological Centre Tam Anh General Hospital Hanoi Vietnam; ^4^ Lung Department University of Medicine and Pharmacy, Vietnam National University Hanoi Vietnam

**Keywords:** cystic, rare disease, tuberculosis cystic lung diseases, tuberculosis

## Abstract

Cystic pulmonary tuberculosis is a unique form of pulmonary tuberculosis (PTB) presenting with multiple reversible cysts of the lung. Unlike the other forms, this cystic lung disease can improve with prompt tuberculosis treatments. Here we report the case of a 15‐year‐old girl who presented with respiratory failure and severe lung damage at hospital admission. We diagnosed her with PTB based on her positive GeneXpert result test. The patient was treated with a standard tuberculosis regimen for 6 months and recovered completely.

## Graphical abstract

We report a rare case of cystic tuberculosis with an unexpectedly rapid and complete improvement following standard TB treatment.

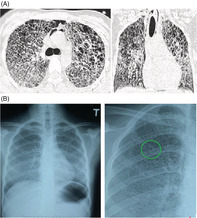

## INTRODUCTION

Pulmonary tuberculosis is known to present itself within a spectrum of different histological and radiological manifestations with various forms caseating or non‐caseating tissue damage. Cystic pulmonary tuberculosis has been reported in the literature only rarely.^1^, ^2^ This form of the disease is characterized by multiple cysts, mostly on the lungs' middle and upper lobes, whose formation can be reversed following treatment with TB regimens.^3^ We report a case of cystic tuberculosis with an unexpectedly rapid and complete improvement following standard TB treatment.

## CASE REPORT

A 15‐year‐old female with no particular medical history was admitted to our hospital with manifestations of hypoxemia, difficult breathing and prolonged fever. She had dyspnea, fever, night sweats and dry cough for 1 month and had been treated at another hospital with Imipenem – Cilastatin and Ciprofloxacin for 10 days with a diagnosis of respiratory failure/severe pneumonia, yet her symptoms worsened with increasing dyspnea and fever and she was then sent to our hospital.

On admission, her temperature was 39°C with a pulse rate of 140 beats/min, a blood pressure of 120/80 mmHg and SpO_2_ of 88% (with an oxygen flow rate of 15 L/min given by mask). Arterial blood gas results were pH 7.36, pCO_2_: 40 mmHg, pO_2_: 89 mmHg. The WBC count was 14,000/mm^3^. Other inflammation markers were elevated as well (CRP: 40 mg/L, Procalcitonin: 0.3). Kidney function and liver function markers were unremarkable. A serological test for HIV was negative.

**FIGURE 1:**
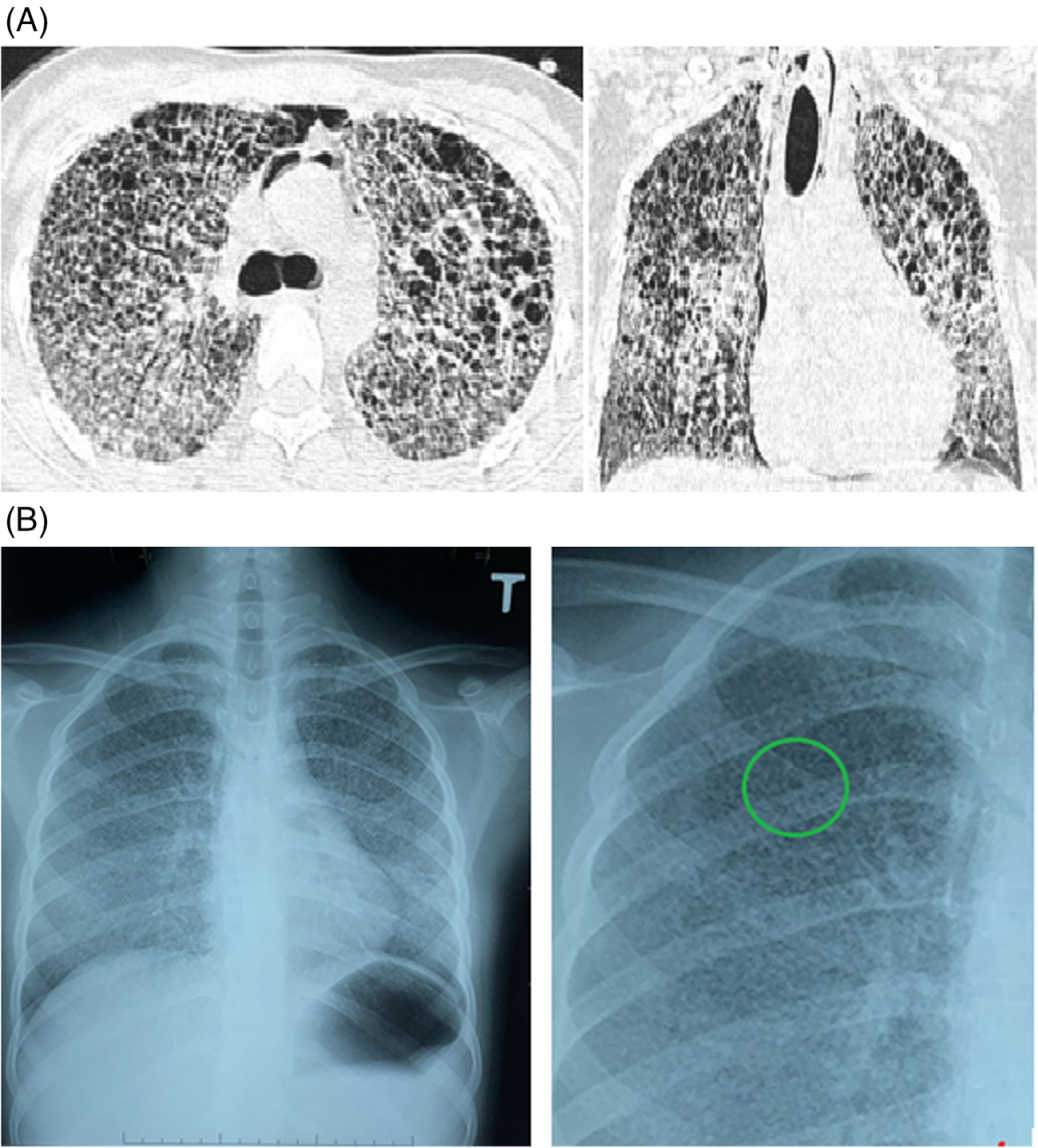
(A) The patient's initial HRCT was taken right after admission with multiple bilateral cystic‐like lesions, predominance in the upper lobes and right middle lobes. (B) Patient's chest x‐ray (2 weeks before hospital admission) with diffuse miliary nodules and scattered cystic changes nodules (circle)

An initial high‐resolution CT (HRCT) taken right after admission found multiple bilateral small cystic‐like lesions, predominantly in the upper and right middle lobes as well as a mild pneumomediastinum (Figure 1A). Almost no normal lung parenchyma was observed in the upper lobes of the two lungs. There was a diffuse increase in lung density in the right lower lobe and a patchy hyperdensity on the left lower lobe.

For further diagnostic hints, we reviewed the patient's earlier X‐ray films. Her chest X‐ray revealed numerous miliary nodules on both lung fields, some of which showed signs of cystic transformation (Figure 1B). Such rapid transformations and progressions of lung damage on X‐Ray and HRCT films were not considered compatible with interstitial lung disease and lead us to look for TB or other acute infectious causes, including bacterial, fungal and viral organisms. The patient was treated with high flow oxygen (HFNC) at 30 L/min and antibiotics while waiting for the result of the TB diagnostic tests. Since the patient's respiratory failure did not allow us to perform routine TB investigations such as bronchoalveolar lavage or sputum testing, multiple GeneXpert gene amplifications (Cepheid, USA) using gastric fluid were done. *Mycobacterium tuberculosis* (Mtb) was found at very low levels in four samples. Cerebral MRI and CSF investigations for neurological TB involvement were normal. A diagnosis of respiratory failure from interstitial lung disease associated with tuberculosis was made and treatment with corticosteroids and the standard TB drugs rifampicin (R), isoniazid (H), ethambutol (E) and pyrazinamide (Z) (known as the 2RHZE/4RHE regimen) was initiated, allowing rapid improvement of the respiratory failure.

**FIGURE 2:**
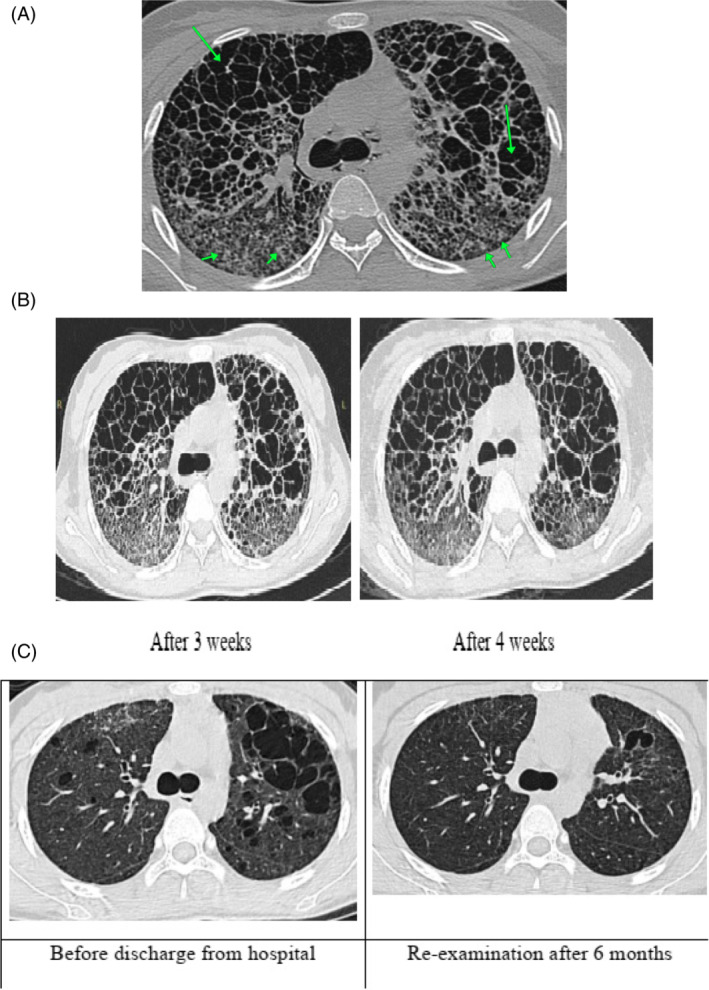
(A) Chest HRCT taken 1 week after initial treatment. Cystic lesion of upper lobes was markedly increased in size with arrangement suggestive of panacinar emphysema (green arrows, with arrowhead point to centrilobular vessel). Several clustered micronodules and microcystic lesions are seen in the hyperdensity regions of the lower lobes. (B) HRCT films were taken 3 and 4 weeks after initial treatment. The cystic lesion of both upper lobes continued to increase in size, some of which fused together to become bullous emphysema. Cystic lesions of lower lobes also increased in number and size. (C) HRCT films taken before discharge and at re‐examination after discharged 6 months. Most of the bullous has been resolved and lung parenchyma restored to normal.

An HRCT was done weekly to evaluate treatment efficiency and progression/ reversion of lung injuries showing that the cystic lesion of upper lobes markedly increased in size over time with several sites displaying tissue arrangements suggestive of bullous emphysema (Figure 2A–C).

After 7 weeks of treatment, despite lung damage appearing still severe on HRCT, the patient was able to take care of herself and perform light activities. The patient's lung status was continued to be observed monthly via HRCT for the next 3 months until discharge. In the fifth month's treatment, overall treatment results were considered very impressive as most areas of bullous tissue had resolved.

Moreover, 6 months after discharge, lung parenchyma appeared restored to normal (Figure 2D).

## DISCUSSION

Cystic and bullous lung tissue changes caused by tuberculosis are an exceedingly rare condition with only a limited number of cases reported. This special form of TB infection features rapidly developing multiple cystic‐like lesions in both lungs, mostly in the upper lobes, along with severe clinical symptoms such as respiratory distress and/or recurrent pneumothorax, leading often to a fatal outcome.

Fortunately, unlike other cystic diseases, proper diagnosis and treatment are able to reverse these lesions completely.

The mechanism of developing cystic pulmonary disease caused by tuberculosis has been discussed in previous reports.^2^, ^4^ The development of granulomatous inflammation can result in a check‐valve type of obstruction and form multiple cysts in the parenchyma, or tubercle rupture and caseation necrosis can lead to interstitial air leakage. The progression of lesions over time in our case strongly supports this check‐valve obstruction hypothesis as the disease mechanism.^4^, ^5^

Chest X‐rays and HRCT are usually the first investigations ordered but are leading often to non‐specific findings that must be differentiated from many other conditions. Imaging quality can also be affected by the patient's clinical condition at the examination as in our case.

For multiple small cystic‐like lesion, several differential diagnoses should be considered, including lymphangioleiomyomatosis, Langerhans‐cell histiocytosis, lymphocytic interstitial pneumonia or *Pneumocystis jirovecii* pneumonia.

Acute respiratory distress syndrome (ARDS) is sometimes reported with the presentation of pulmonary TB with a probability of around 5%. In such reports, HRCT findings included diffuse bilateral opacities, similar to our case. However, previous cases featured respiratory failure requiring intubation and assisted ventilation, followed by problematic weaning processes and secondary infectious. In our case, we decided to provide only antituberculosis therapy together with HFNC, to prevent coinfections by minimizing invasive procedures.

Our patient is one among the survivors with a remarkable recovery from tuberculosis with standard treatments despite severe lung damage.^4^, ^5^ This result has likely been achieved on the basis of the TB treatment initiation immediately following availability of the GeneXpert test results, a well‐established routine practice in Vietnam. Indeed, this rapid treatment start contrasts with other reports on cystic tuberculosis where treatment was delayed pending results from smear microscopy or mycobacterial cultures.

## AUTHOR CONTRIBUTION

Luong Dinh Van, Huy Le Ngoc and Ngoc Nguyen Thi Bich took part in establishing the diagnosis of this patient and the drafting and supervision of this case report. Thinh Nguyen Hoang participated in the radiological studies and the quality assurance of the imaging. Michel Pletschette conceived the format of the article and together with Anh Nguyen Tuan's contribution did the final draft and revision.

## CONFLICT OF INTEREST

None declared.

## ETHICS STATEMENT

The authors declare that appropriate written informed consent was obtained for the publication of this manuscript and accompanying images.

## Data Availability

The data that support the findings of this study are available on request from the corresponding author. The data are not publicly available due to privacy or ethical restrictions.
